# Timing of diagnosis, depression and self-harm in adolescents with autism spectrum disorder

**DOI:** 10.1177/1362361320945540

**Published:** 2020-08-08

**Authors:** Mariko Hosozawa, Amanda Sacker, Noriko Cable

**Affiliations:** 1 University College London, UK; 2 Juntendo University, Japan

**Keywords:** adolescence, autism spectrum disorder, depression, diagnosis, self-harming behaviour

## Abstract

This exploratory study aimed to explore the association between depression and self-harming behaviour in adolescence and the timing of diagnosis for autism spectrum disorder. We analysed data on 11,320 14 year olds, including 396 children with autism spectrum disorder, from the UK Millennium Cohort Study. Exposures were the timing of diagnosis for autism spectrum disorder confirmed by parental report at ages 5, 7, 11 and 14. Outcomes were depression measured by the Short Mood and Feelings Questionnaire (scores ⩾12) and self-report of self-harming behaviour, both assessed at age 14. Data were analysed using multivariable regression analysis. 91% of the autism spectrum disorder group had within-typical-range cognitive ability. After adjusting for child and family confounders, there was a positive linear trend between diagnostic age and depression, with children diagnosed between ages 7 and 11 and children diagnosed after age 11 showing increased symptoms (odds ratio = 2.21 and 3.58, respectively). A similar trend was observed for self-harming behaviour, with children diagnosed after age 11 showing the strongest association (odds ratio = 3.16). These results suggest the importance of earlier diagnosis in preventing secondary mental health problems in this population, particularly among those without cognitive delays. Further studies replicating across a wider intellectual spectrum and clarifying the underlying mechanism are warranted.

**Lay Abstract:**

Children with autism spectrum disorder are at increased risk of depression and self-harming behaviours. The question of whether timing of diagnosis of autism spectrum disorder is associated with these consequences in adolescence has not yet been studied. This exploratory study aimed to explore the association between depression and self-harming behaviour in adolescence and the parent-reported timing of diagnosis for autism spectrum disorder using a large population-based cohort in the United Kingdom. Most of the children with autism spectrum disorder in our study had within-typical-range cognitive ability. We found a linear association between timing of autism spectrum disorder diagnosis and depression and self-harming behaviour in adolescence; later diagnosis of autism spectrum disorder, particularly diagnosis in adolescence, was associated with the increased risk of self-reported depressive symptoms and self-harming behaviour in adolescence among children with autism spectrum disorder. Our findings, albeit observational, suggest that interventions targeting the earlier diagnosis of autism spectrum disorder and approaches to improve person–environment fit may help prevent secondary mental health problems in this population, particularly among those without cognitive delays and those diagnosed late. Further studies replicating across a wider intellectual spectrum and clarifying the underlying mechanism are warranted.

Individuals with autism spectrum disorder (ASD) are at increased risk of depression. Studies have shown the point prevalence of depression in children with ASD to be as high as 26% and that it may increase during adolescence (Davignon et al., 2018; DeFilippis, 2018; Gotham et al., 2015; Rai, Culpin, et al., 2018; Rai, Heuvelman, et al., 2018; Simonoff et al., 2008). Recent studies have reported high rates of self-harming behaviour including suicidal attempt, ideation and completion in this population (Davignon et al., 2018; Hedley et al., 2018; Segers & Rawana, 2014). Depression and self-harming behaviour do not only have a negative impact on the quality of life of individuals with ASD but are also important risk factors for suicidal death, a leading cause of premature mortality in this population (Hirvikoski et al., 2016). Therefore, finding potentially modifiable factors to prevent or reduce depression and self-harming behaviours in children with ASD is of clinical importance.

Previous research into risk and protective factors for depression and self-harming behaviour among young people with ASD has mainly focused on individual factors (e.g. gender, age, cognitive level, family history of depression and quality of social relationships; DeFilippis, 2018; Gadow et al., 2008; Hedley et al., 2018; Rai, Heuvelman, et al., 2018; Simonoff et al., 2008; Soke et al., 2018). However, based on the theoretical framework of developmental psychopathology, mental disorders are not simply a characteristic of the individual but are the result of a mismatch between the individual and their environment (Rutter & Sroufe, 2000). Therefore, considering contextual factors which may increase or decrease the risk for depression and self-harming behaviour in this population could be a potential area of research for prevention.

Earlier identification of ASD has been proposed as improving the long-term outcomes of ASD such as social communication skills, language ability and adaptive behaviours through early intervention (Elder et al., 2017). A previous study among adults diagnosed as Asperger’s syndrome suggested the importance of late diagnosis being a potential risk factor for suicidal behaviours (Cassidy et al., 2014); however, the question of whether earlier identification of ASD could reduce the risk of developing depression and self-harming behaviour in adolescence in children with ASD has not been studied. Based on the framework above, the earlier identification of ASD may improve ‘person–environment fit’ and decrease the risk of depression and self-harming behaviour. Given the evidence that nearly half of children with ASD are diagnosed over age 5 in the United Kingdom (Brett et al., 2016), it is important to understand the association between timing of diagnosis and depression and self-harming behaviour in adolescence in children with ASD.

Using data from a large population-based cohort in the United Kingdom, we asked whether the timing of diagnosis is associated with mental health problems in adolescence among children with ASD. We hypothesized that the timing of diagnosis would be positively associated with the presence of depression and self-harming behaviour at age 14.

## Methods

### Study population

Data came from the Millennium Cohort Study (MCS), a nationally representative birth cohort study which included 19,517 children born in the United Kingdom between September 2000 and January 2002. Data were collected when the cohort members were 9 months and 3, 5, 7, 11 and 14 years old. The study used a stratified cluster design to oversample children living in disadvantaged areas and those living in areas with a high proportion of ethnic minority groups. Details of the survey design, recruitment process and fieldwork have been described elsewhere (Connelly & Platt, 2014). Of the 11,872 children who took part in the MCS at age 14 when the information on our main outcomes was measured, we used the data from 11,320 children with valid information on our explanatory (i.e. diagnosis of ASD) and at least one of our main outcome variables (i.e. depressive symptoms and self-harming behaviour). Data were obtained via the UK Data Archive http://doi.org/10.5255/UKDA-SN-8156-5. Further information about the study is found at https://cls.ucl.ac.uk/cls-studies/millennium-cohort-study/.

### Parent-reported diagnosis of ASD

A diagnosis of ASD was confirmed by asking the parents at each sweep from age 5 the following question: ‘Has a doctor or other health professional ever told you that your child had autism, Asperger’s syndrome or another autistic spectrum disorder?’ This question has been used to ascertain ASD in other population-based studies (Kogan et al., 2009) and a child whose parent responded ‘yes’ to this question at any of the four sweeps was identified as having an ASD diagnosis. A five-category variable was created using the age of the sweep at which the parent first reported their child having a diagnosis of ASD: (0) no diagnosis; (1) reporting diagnosis at age 5 interview; (2) age 7; (3) age 11 interview; and (4) age 14.

### Depressive symptoms

Depressive symptoms were measured using the Short Mood and Feelings Questionnaire (SMFQ) which was administered at age 14 (Angold et al., 1995). The SMFQ is a 13-item instrument with a total score ranging from 0 to 26. It is a well-validated self-report questionnaire for children and adolescents to evaluate depressive symptoms in the 2 weeks prior (Thabrew et al., 2018; Thapar & McGuffin, 1998), which has been used in adolescents with ASD (Mazefsky et al., 2014; Rai, Heuvelman, et al., 2018). We included up to 1-item missing for this scale. In our study sample, the SMFQ showed high internal consistency both in the general population and in the ASD group (alpha = 0.93 for the No ASD group, 0.92 for the ASD group). In addition to the raw total scores of the SMFQ, a binary variable was created to capture the no versus depressed status. For this, we used a cut-off score of 12 or more as having ‘depression’, based on a previous study which investigated the reliability and validity of the SMFQ (sensitivity 84.2%, specificity 68.2%; Thabrew et al., 2018).

### Self-harming behaviour

Self-harming behaviour was assessed based on the response to a question asked at age 14: ‘In the past year have you hurt yourself on purpose in any way?’ Participants who answered ‘Yes’ to this question were classified as having experience of self-harming behaviour.

### Covariates

We included the following variables, all measured at the age 5 interview when the diagnosis of ASD was asked about for the first time in the MCS, as potential confounding factors: child sex, multiple birth indicator, cognitive ability (obtained from the mean score of three subscales of the British Ability Scales (BAS) II administered at age 5: the BAS II Naming Vocabulary Subscale indicative of the level of the spoken vocabulary, the BAS II Picture Similarity Subscale indicative of problem-solving abilities and the BAS II Pattern Construction Subscale indicative of spatial awareness (Elliott et al., 1997). Each score was standardized after adjusting for age and difficulty, and those scoring 1 SD below the standardized mean were defined as having low cognitive ability), highest parental education (attaining advanced level or higher, a qualification required to enter a university or higher), relative income poverty of the household (indicated by household equivalized income of less than 60% of the UK national median household income; Agalioti-Sgompou et al., 2017) and parental depression (defined as depressed when scoring 13 or more on the Kessler-6 scale; Prochaska et al., 2012). We also included parent-rated emotional symptoms obtained from the emotional symptoms subscale of the Strengths and Difficulties Questionnaire (SDQ; range 0–10; higher scores indicate more severe emotional symptoms).

### Data analysis

We first explored the differences between children who were included in our study (i.e. the analytic sample, *N* = 11,320) and those who were excluded (i.e. the non-analytic sample, *N* = 8197) for the two diagnostic groups. Next, we conducted a bivariate analysis to examine the relationship between variables. We then examined the association between the timing of diagnosis for ASD and depression and self-harming behaviour using multivariable linear and logistic regression analysis. An unadjusted model (model 1), a model adjusted for sex (model 2), and a model further adjusted for multiple birth status, family covariates, cognitive ability and emotional symptoms of the child (model 3) were examined. In this analysis, the no-ASD group was taken as the reference category to increase statistical precision. Differences in associations by sex in the multivariable analysis were examined using an interaction term. There was little evidence of effect modification by sex. Therefore, data from both sexes were combined and analysed together. All the analyses were weighted using weights supplied by the MCS to take into account the stratified sample, oversampling of subgroups and attrition. Analyses were conducted using Stata version 15 (Stata Corp, College Station, TX).

### Sensitivity analysis

To test the robustness of the association between timing of diagnosis and depression and self-harming behaviour, we conducted the following three sensitivity analysis: (1) We further adjusted for a score indicating the level of ASD-related behaviours which is a combination of items from age 5 and 7 interviews (higher scores indicate greater impairment, mainly in social communication) (Russell et al., 2015). This score comprises the teacher evaluated ‘social development’ and ‘language for communication’ subscales from a questionnaire which mimicked the Foundation Stage Profile; teachers completed this questionnaire at the end of preschool (i.e. age 5 interview) to assess early learning goals in children (Johnson, 2008), and parents and teacher-reported items from the SDQ completed at the age 7 interview. All the items were standardized then added to create a composite score (calculated where data were available for at least five of the eight items). We also replaced the outcome ‘depression’ by a continuous score using the number of total depressive symptoms reported (range, 0–26) for this analysis. (2) We modelled the outcome ‘parent-rated emotional symptoms’ measured by the emotional symptoms subscale of the SDQ when the child was around age 14 to examine whether the association between timing of diagnosis and mental health problems is similar across informants (i.e. children’s self-report and parents’ report). (3) We calculated the magnitude of an unobserved confounder that would explain away the main effect of exposures on outcomes using a method outlined by VanderWeele and Ding (2017).

### Missing data

The proportion of data that were missing in our study sample varied by variable from 5.7% on parental education to 10.9% on parental depression (18.5% for the level of ASD-related behaviours in the sensitivity analysis). Missing data were imputed using multiple imputation by chained equations and regression analyses were run across 20 imputed data sets to calculate overall estimates (Madley-Dowd et al., 2019; I. R. White et al., 2011). The imputed results were broadly similar to those obtained using observed cases (Supplemental Table S1) and therefore the former are presented here.

### Patient and public involvement

Participants of the MCS were not involved in setting the research question or the outcome measures, nor were they involved in the design or implementation of the study. No participants were asked to advise on the interpretation or writing of the results. However, the results are disseminated to study participants through their dedicated website: https://childnc.net/.

## Results

The differences between the analytic sample (i.e. children who were included in this study) and the non-analytic sample (i.e. those who ever participated in the MCS but were not included in this study) by the presence of a diagnosis of ASD are shown in Supplemental Table S2. In children with a diagnosis of ASD, children who were not included in this study had more cognitive delay, lower parental education, higher ASD-related behaviours and were more likely to be from the earlier diagnosed groups (i.e. Age 5 and Age 7 groups); however, the two samples did not differ significantly on parent-rated emotional symptoms at age 5.

The characteristics of our analytic sample by the timing of ASD diagnosis are presented in Table 1. In our analytic sample, 396 children received a diagnosis of ASD by age 14. Compared to the No ASD children in the study, children in the ASD groups had a significantly higher level of ASD-related behaviours and parent-rated emotional symptoms at around age 5 across the diagnostic-age groups (*p* < 0.001 for all comparisons). Girls with ASD were likely to be diagnosed later. Most of the children with ASD (ranging from 88.5% to 91.8% depending on diagnostic-age group) had their cognitive ability within the typical range, and this rate did not differ significantly among all groups.

**Table 1.** Characteristics of multiply imputed sample by the timing of diagnosis for autism spectrum disorder (ASD, *N* = 11,320).

	No ASD (0) *n* = 11,307	ASD (*n* = 396)					*p* ^a^	Significant group difference^b^
		Age 5 (1) *n* = 61	Age 7 (2) *n* = 65	Age 11 (3) *n* = 138	Age 14 (4) *n* = 132	Total *n* = 396		
	%	%	%	%	%	%		
Sex of the child							<0.001	0 vs 1,2,3,4 1 vs 3,4
Male	50.2	91.2	77.2	79.5	75.0	79.3		
Female	49.8	8.8	22.8	20.5	25.0	20.7		
Cognitive ability							0.12	–
Within typical range	96.0	89.0	88.5	91.8	91.7	90.8		
Below 1 SD	4.0	11.0	11.5	8.2	8.3	9.2		
Parental highest education^c^							0.44	–
A-level or above	43.1	32.7	45.5	44.2	35.7	39.4		
Below A-level	56.9	67.3	54.5	55.8	64.3	60.6		
Low household income^d^							0.002	0 vs 2,4
No	64.4	58.0	48.9	55.3	46.2	51.4		
Yes	35.6	42.0	51.1	44.7	53.8	48.6		
Parental depression							0.43	–
No	84.1	90.9	84.9	82.0	78.5	82.6		
Yes	15.9	9.1	15.1	18.0	21.5	17.4		
Parent-rated emotional symptoms at age 5, mean (SE)	1.4 (0.0)	2.6 (0.3)	2.9 (0.4)	1.9 (0.2)	2.4 (0.2)	2.4 (0.1)	<0.001	0 vs 1,2,3,4 1,2 vs 3
Level of ASD-related behaviours, mean (SE)^e^	–0.2 (0.1)	7.3 (0.9)	6.9 (1.0)	5.4 (0.6)	4.2 (0.6)	5.5 (0.4)	<0.001	0 vs 1,2,3,4 1,2 vs 4

ASD: autism spectrum disorder; SD: standard deviation; SE: standard error..

Weighted percentages are shown. Variables are measured at age 5 interview of the study unless otherwise mentioned.

a *p*-value for group difference (i.e. No ASD, Age 5, 7, 11 and 14 groups) obtained using Stata command ‘contrast’ which performs analysis of variance style test of main effects and post hoc tests.

b Significant difference was defined as a difference at 5% levels.

c A-levels are final examinations usually taken at age 18 before university entrance.

d Below 60% median of UK national household income.

e A score indicating the level of ASD-related behaviours measured from teacher and parent reports when the child was around age 5 to 7.

### The occurrence of depression and self-harming behaviour

**Figure 1.**
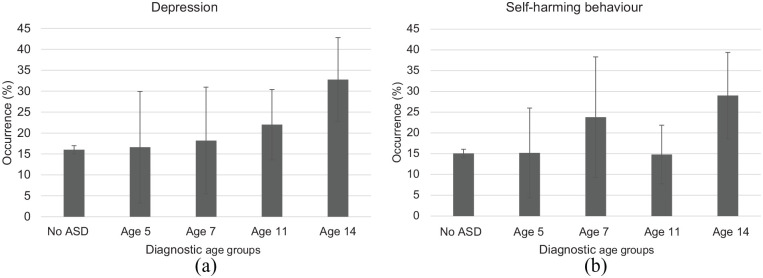
Unadjusted prevalence of (a) depression and (b) self-harming behaviour at age 14 by the timing of diagnosis for autism spectrum disorder. The occurrence increased in a linear trend with increasing diagnostic age for depression (*p*_trend_ = 0.003) and for self-harming behaviour (*p*_trend_ = 0.03).

The occurrence of depression and self-harming behaviour at age 14 is shown in Figure 1. Children who were diagnosed after age 11 (Age 14 group) showed the highest rate of depression and self-harming behaviour at age 14. There was a trend of increased risk of depression with later diagnosis (*p* for linear trend = 0.003). For self-harming behaviour, the same linear trend was observed though the association was weaker (*p* for linear trend = 0.03), with children diagnosed between age 5 and 7 (Age 7 group) showing a higher prevalence of self-harming behaviour than those diagnosed between age 7 and 11 (Age 11 group).

The relationship between the timing of diagnosis and the outcomes was further confirmed by multivariable regression analysis (Table 2). After adjusting for confounders, being diagnosed after age 7 (Age 11 group and Age 14 group) was associated with depression (odds ratio [OR] = 2.21, 95% confidence interval [CI] = 1.27–3.83; OR = 3.58, 95% CI = 2.13–5.96, respectively). Being diagnosed between age 5 and 7 (Age 7 group) and being diagnosed after age 11 (Age 14 group) were significantly associated with self-harming behaviours (OR = 2.36, 95% CI = 1.10–5.07; OR = 3.16, 95% CI = 1.82–5.45, respectively). Children diagnosed before age 5 were the only group that was not significantly associated with either depression or self-harming behaviour (OR = 1.78, 95% CI = 0.67–4.75 for depression; OR = 1.63, 95% CI = 0.66–3.98 for self-harming behaviour).

**Table 2.** Depression and self-harming behaviour in adolescence by the timing of diagnosis for autism spectrum disorder.

	Unadjusted occurrence, %^a^	Model 1 (Unadjusted)		Model 2 (Sex adjusted)		Model 3 (Fully adjusted^b^)	
	Mean (SE)	OR	95% CI	OR	95% CI	OR	95% CI
Depression							
No ASD	16.0 (0.5)	1 (ref)	–	1 (ref)	–	1 (ref)	–
Age 5	16.6 (6.8)	1.05	0.40–2.76	1.85	0.69–4.96	1.78	0.67–4.75
Age 7	18.2 (6.5)	1.17	0.49–2.78	1.67	0.74–3.77	1.56	0.70–3.49
Age 11	22.0 (4.3)	1.49	0.91–2.44	2.23	1.29–3.85	2.21	1.27–3.83
Age 14	32.8 (5.1)	2.57	1.62–4.09	3.73	2.24–6.23	3.58	2.13–5.96
*p* for linear trend	0.003	0.002		<0.001		<0.001	
Self-harming behaviour							
No ASD	15.1 (0.5)	1 (ref)	–	1 (ref)	–	1 (ref)	–
Age 5	15.2 (5.5)	1.01	0.43–2.35	1.80	0.76–4.22	1.63	0.66–3.98
Age 7	23.8 (7.4)	1.76	0.79–3.93	2.58	1.20–5.59	2.36	1.10–5.07
Age 11	14.8 (3.6)	0.98	0.56–1.73	1.45	0.80–2.64	1.44	0.79–2.61
Age 14	29.0 (5.3)	2.30	1.37–3.84	3.33	1.92–5.77	3.16	1.82–5.45
*p* for linear trend	0.03	0.03	–	0.005		0.006	

OR: odds ratio; SE: standard error; CI: confidence interval; ASD: autism spectrum disorder.

a Weighted percentages are shown.

b Additionally, adjusted for multiple birth, parental education, household income, parental depression, cognitive ability, and parent-rated emotional symptoms at age 5.

A sensitivity analysis demonstrated that the linear association between timing of diagnosis and depression and self-harming behaviour did not change after adjusting for the level of ASD-related behaviours at around age 5 to 7 and when depression was treated as a continuous variable (Supplemental Table S2). When we modelled parent-rated emotional symptoms at age 14, a similar linear association between the timing of diagnosis and parent-rated emotional symptoms was obtained after adjusting for confounders and for the level of ASD-related behaviours (*p* for linear trend < 0.001, Model 2, Supplemental Table S3). Finally, our sensitivity analysis for unmeasured confounders revealed that a substantial amount of unmeasured confounding would be necessary to explain away the observed exposure–outcome associations (Supplemental Table S4). For example, considering the association between diagnosis for ASD after age 11 (Age 14 group) and depression, an unmeasured confounder associated with both the exposure and the outcome with an odds ratio of 6.62 or more would be needed for the timing of diagnosis to have no true association with depression.

## Discussion

Using data from a representative cohort of UK children, we explored whether the timing of diagnosis for children with ASD was associated with depression and self-harming behaviour in adolescence. After adjusting for confounders including sex, family socio-economic status, family depression, cognitive ability and emotional symptoms at around age 5 of the child, the occurrence of depression and self-harming behaviour increased linearly with diagnostic-age period; children diagnosed before age 5 did not show any significant association with the outcomes, and children diagnosed after age 11 showed the strongest association with both outcomes. Our sensitivity analysis confirmed that parent-rated emotional symptoms at age 14 also increased linearly with age of diagnosis.

To our knowledge, this study is the first to explore the association between the timing of diagnosis and mental health in children with ASD in adolescence. Our results support our a priori hypothesis that later diagnosis would be associated with increased mental health problems in adolescence in children with ASD. Our findings, albeit observational, extend empirical support for the value of early diagnosis for children with ASD and highlight the importance of considering environmental factors and approaches to improve person–environment fit in children with ASD to prevent secondary mental health problems in this population.

Several underlying mechanisms might explain the observed association in our study. First, previous studies have shown that earlier diagnosis is associated with early intervention and increased evidence-based treatment use (Zuckerman et al., 2017), which may improve the child’s social and coping skills, leading to reduced mental health problems in adolescence. Second, early diagnosis is known to be associated with adequate support (e.g. special educational support) at school (Mandell et al., 2005), which is also known to reduce later mental health problems in this population (Gadow et al., 2008). Third, a previous longitudinal study has demonstrated the mediating effect of bullying-victimization on depression in children with ASD (Rai, Culpin, et al., 2018). It may be that increased exposure to bullying-victimization resulting from later diagnosis could partly explain the increased depression and self-harming behaviour. Although we cannot provide longitudinal evidence for the above hypothesis, future research investigating the underlying mechanisms of the observed association in this study could offer additional evidence for improved interventions and policies to reduce depression and self-harming behaviour and support a successful transition into adulthood for children with ASD.

### Strength and limitations

One strength of this study is the relatively large number of children with ASD drawn from a population sample born around the millennium. The inclusion of non-ASD children allowed for more precise group comparisons, while the rich prospectively measured data allowed us to adjust for a range of relevant confounders. We used self-reports by adolescents for our outcomes, and our use of parent-rated emotional symptoms in our sensitivity analysis strengthened our result by showing that a similar association was observed across informants.

Our study has several limitations. First, our sample size was relatively large for studies of this population; however, the small number of girls with ASD in our study likely limited our power to examine interaction effects indicating sex differences in the outcomes. Second, the diagnosis of ASD was based on a parental report which has not been externally validated in the MCS. However, parental report on the child’s ASD diagnosis has been used in many other population-based studies and has been proven to have good reliability (Daniels et al., 2012; Kogan et al., 2009). We were not able to identify the ‘current status’ of ASD diagnosis, because in the later sweeps (i.e. from age 11 interview) the MCS asked the diagnostic status of ASD only to those who did not report a prior diagnosis of ASD. As children who lost their ASD diagnosis were included in the ASD groups in our study, it could have downwardly biased estimates of mental health problems. However, it has been reported in a previous study that both children who lost their parent-reported ASD diagnosis and children with a current diagnosis had similarly high rates of mental health problems including depression when compared to those who never reported an ASD diagnosis (Kogan et al., 2009). Third, despite its use for adolescents with ASD (Mazefsky et al., 2014; Rai, Heuvelman, et al., 2018), the SMFQ has not been validated among adolescents with ASD and could be a source of measurement error. However, our sensitivity analysis using parent-rated emotional symptoms confirmed similar trends across informants, thus supporting our findings. Forth, although the effect of severity of ASD on psychiatric symptoms has been inconsistent in previous studies (Hedley et al., 2018; Simonoff et al., 2008), we did not have information on the severity of ASD symptoms aside from a score indicating levels of ASD-related behaviour at around ages 5 to 7. Some may query whether those children diagnosed latest were likely to have less severe ASD-related symptoms and were thus only identified when they presented mental health problems. However, our descriptive analysis demonstrates that the level of ASD-related behaviours at around age 5 to 7 did not differ significantly among children diagnosed after age 7 (i.e. Age 11 and 14 groups). Furthermore, our sensitivity analysis accounting for the level of ASD-related behaviours confirmed that the association between timing of diagnosis and depression and self-harming behaviour was independent of the level of ASD-related behaviours at around age 5 to 7, suggesting that reverse causation is unlikely to account for the association we found. Fifth, we minimized potential selection bias from missing cases by applying survey weights and multiple imputation for missing covariate data; however, our sample bias analysis showed that those with cognitive delay, higher ASD-related behaviours at around age 5 to 7 and from the earlier diagnosed groups (i.e. Age 5 and 7 groups) were more likely to be excluded from our sample. This could have led to an overestimation of mental health problems particularly in the earlier diagnosed groups because previous studies demonstrated that the risk of depression and self-harming behaviour in people with ASD is higher in those without intellectual disability (Rai, Heuvelman, et al., 2018; Soke et al., 2018). Contrary to expectation, our sample bias analysis did not find a significant difference regarding rates of low household income between children with ASD who were included in the study and those who were excluded. A previous study reported that children from low household income families are more likely to be diagnosed late (Hosozawa et al., 2020), which may explain this finding. Finally, in our sample, the cognitive ability of around 90% of the children with ASD was within the normal range, which is higher than a previously reported figure for children with ASD (Baio et al., 2018). Therefore, the applicability of our findings is likely to be limited to children with ASD with higher intellectual abilities who could answer self-report questionnaires and not the entire spectrum of children with ASD. Nevertheless, the former group is known to be more vulnerable to mental health problems as noted above (Rai, Heuvelman, et al., 2018), making our findings important despite this limitation. Future studies replicating our results in children with ASD with a wider intellectual spectrum would be helpful in this regard.

Although we found a general linear trend between increased diagnostic age and increased prevalence of self-harm, children who were diagnosed between ages 5 and 7 (Age 7 group) showed a higher prevalence of self-harming behaviour than those diagnosed between ages 7 and 11 (Age 11 group). Although we cannot give explicit explanations for this finding, it may be that the child’s characteristics other than autism, for example, impulsiveness or anxiety, which has been reported as risk factors for self-harming behaviour in this population (Licence et al., 2019; Moseley et al., 2019; S. W. White et al., 2009), may have contributed to the result. Considering the effect of pre-existing comorbid psychiatric conditions would be important in future research to detect those who are at most risk of developing depression and self-harming behaviour in children with ASD.

## Conclusion

In summary, we report that a later diagnosis of ASD is associated with increased risk of depression and self-harming behaviour in adolescence, particularly among those without cognitive delays. Although further studies clarifying the underlying mechanism are needed, the results are consistent with the suggestion that interventions targeting the earlier diagnosis of ASD and approaches to improve person–environment fit, particularly in those diagnosed after primary school, may help reduce secondary mental health problems in adolescence. Furthermore, it may be important to carry out assessments for comorbid mental health states when clinicians see late-diagnosed children with ASD. Future studies replicating the association across a wider intellectual spectrum of children with ASD are also warranted.

## Supplemental Material

hosozawa_suptables-asddep0428 – Supplemental material for Timing of diagnosis, depression and self-harm in adolescents with autism spectrum disorder Click here for additional data file. Supplemental material, hosozawa_suptables-asddep0428 for Timing of diagnosis, depression and self-harm in adolescents with autism spectrum disorder by Mariko Hosozawa, Amanda Sacker and Noriko Cable in Autism
