# Supplementary material for: Timing of diagnosis, depression and self-harm in adolescents with autism spectrum disorder
Source: Autism. 2020 Aug 8;25(1):70–8. doi: 10.1177/1362361320945540 (PMC8162135; doi:10.1177/1362361320945540)
Supplement: hosozawa_suptables-asddep0428 – Supplemental material for Timing of diagnosis, depression and self-harm in adolescents with autism spectrum disorder [file hosozawa_suptables-asddep0428.pdf]

Supplementary Table S1 *Characteristics of the observed sample by the timing of diagnosis for autism spectrum disorder (ASD, N = 11,320)*

	No ASD (n = 10,924)		ASD (n = 396)							
			Age 5 (n = 61)		Age 7 (n = 65)		Age 11 (n = 138)		Age 14 (n = 132)	
	n	%	n	%	n	%	n	%	n	%
Sex of the child										
Male	5,300	50.3	54	91.2	52	77.2	104	79.5	91	75.0
Female	5,624	49.8	7	8.8	13	22.8	34	20.5	41	25.0
Cognitive ability										
Within typical range	9,861	96.2	40	94.9	54	88.6	109	93.3	113	92.0
Below 1 SD	336	3.8	5	5.1	6	11.4	12	6.7	11	8.1
Parental highest education										
A-level or above	5,501	44.2	29	32.7	33	44.6	67	44.9	59	37.4
Below A-level	4,793	55.9	32	67.3	30	55.4	60	55.1	65	62.7
Low household income										
No	7,176	65.2	44	58.0	36	47.9	78	53.8	74	47.0
Yes	3,085	34.8	17	42.0	27	52.1	49	46.2	50	53.0
Parental depression										
No	9,434	96.5	56	93.7	56	88.5	113	93.4	111	89.3
Yes	289	3.5	3	6.3	6	11.5	8	6.6	9	10.7
Parent-rated emotional symptoms at age 5, mean (SD)	1.4	1.6	2.7	1.8	2.9	2.5	1.8	1.7	2.4	1.7
Level of ASD-related behaviours, mean (SD)	-0.3	3.7	7.2	4.9	6.8	6.6	5.1	5.0	4.0	4.0

Note. Unweighted numbers and weighted percentages are shown. N varies due to missing data.

Supplementary Table S2 *Sample bias analysis by diagnostic groups*

	Total MCS sample (N = 19,517)[a]									
	With diagnosis of ASD (n = 624)					Without diagnosis of ASD[b] (n = 18,893)				
	Analytic sample[c] (n = 396)		Non-analytic sample[d] (n = 228)		P^e	Analytic sample[c] (n = 10,924)		Non-analytic sample[d] (n =7,969)		P^e
	n	%	n	%		n	%	N	%	
Sex of the child					.42					< .001
Male	301	75.6	180	78.9		5,300	48.7	4,238	53.0	
Female	95	24.5	48	21.1		5,624	51.3	3,731	47.0	
Cognitive ability					< .001					< .001
Within typical range	316	92.5	118	80.9		9,861	97.6	4,083	94.5	
Below 1 SD	34	7.5	31	19.1		336	2.4	289	5.5	
Parental highest education					.006					< .001
A-level or above	188	53.4	86	40.6		5,501	57.3	1,820	43.2	
Below A-level	187	46.6	118	59.5		4,793	42.7	2,729	56.8	
Low household income					.95					< .001
No	232	65.4	123	65.0		7,176	76.7	2,613	64.3	
Yes	143	34.7	83	35.0		3,085	23.3	1,892	35.7	
Parental depression					.94					< .001
No	336	92.6	172	92.4		9,434	97.5	4,073	96.1	
Yes	26	7.4	18	7.6		289	2.5	185	3.9	
Parent-rated emotional symptoms at age 5, mean (SD)	2.2	2.0	2.3	2.1	.51	1.3	1.5	1.4	1.6	< .001
Level of ASD-related behaviours, mean (SD)	4.7	5.4	8.3	6.1	< .001	-0.8	3.5	0.3	4.1	< .001
Timing of diagnosis for ASD					< .001					
Age 5	61	14.4	72	31.1		-	-	-	-	
Age 7	65	15.3	61	28.2		-	-	-	-	
Age 11	138	34.6	73	30.9		-	-	-	-	
Age 14	132	35.7	22	9.8		-	-	-	-	

Note. Unweighted numbers (n varies due to missing data) and weighted percentages are shown. [a] The number represents children who ever took part in the MCS. [b] Includes 2,963 children with a missing answer for the diagnosis of ASD. [c] Consists of 11,320 children who were included in this study [d] Consists of 8,197 children who were excluded from this study. [e]*P*-value obtained from chi-square test for categorical variables and Wald test for continuous variables.

Supplementary Table S3 *Differences in mean depressive symptom scores, parent-rated emotional symptoms, and odds of self-harming behaviour by the timing of diagnosis for autism spectrum disorder after adjusting for confounders (Model 1) and further adjusting for the level of ASD-related behaviours (Model 2)*

	Model 1 Confounder adjusted model[a]		Model 2 Further adjusted for level of ASD-related behaviours[b]	
Depressive symptoms	Mean difference	95% CI	Mean difference	95% CI
No ASD	(ref)	-	(ref)	-
Age 5	0.20	-0.29–0.68	0.15	-0.34–0.65
Age 7	0.28	0.02–0.53	0.22	-0.04–0.49
Age 11	0.37	0.20–0.54	0.33	0.15–0.51
Age 14	0.58	0.39–0.78	0.56	0.37–0.75
P for linear trend	< .001	–	< .001	
Self-harming behaviour	OR	95% CI	OR	95% CI
No ASD	1 (ref)	-	1(ref)	-
Age 5	1.63	0.66–3.98	1.47	0.59–3.64
Age 7	2.36	1.10–5.07	2.15	1.00–4.61
Age 11	1.44	0.79–2.61	1.33	0.73–2.44
Age 14	3.16	1.84–5.45	3.00	1.73–5.21
P for linear trend	.006		.008	
Parent-rated emotional symptoms at age 14	Mean difference	95% CI	Mean difference	95% CI
No ASD	(ref)	-	(ref)	-
Age 5	0.77	0.16–1.38	0.08	-0.50–0.66
Age 7	1.66	0.91–2.41	1.06	0.33–1.79
Age 11	1.92	1.51–2.34	1.43	0.99–1.87
Age 14	2.23	1.75–2.71	1.89	1.40–2.37
P for linear trend	< .001		< .001	

Note. ASD: autism spectrum disorder; CI: confidence interval. [a]Adjusted for sex, multiple birth, parental education, household income, parental depression, cognitive ability, and parent-rated emotional symptoms at age 5. [b]A score indicating the level of ASD-related behaviors measured from teacher and parent reports

when the child was around age 5 to 7.

Supplementary Table S4 *Unmeasured confounding sensitivity analysis for the association between depression and self-harming behaviour and the timing of diagnosis for autism spectrum disorder*

	Fully adjusted observed odds ratios, OR_{XY} (95% confidence interval)[a]	Joint minimum strength for unmeasured confounders, OR_{UX} and OR_{UY} [b]
Depression		
No ASD	1 (ref)	-
Age 5	1.78 (0.67–4.75)	2.96, 2.96
Age 7	1.56 (0.70–3.49)	2.50, 2.50
Age 11	2.21 (1.27–3.83)	3.84, 3.84
Age 14	3.58 (2.13–5.96)	6.62, 6.62
Self-harming behaviour		
No ASD	1 (ref)	-
Age 5	1.63 (0.66–3.98)	2.64, 2.64
Age 7	2.36 (1.09–5.07)	4.16, 4.16
Age 11	1.44 (0.79–2.61)	2.23, 2.23
Age 14	3.16 (1.82–5.45)	5.78, 5.78

Note. ASD: autism spectrum disorder, OR: odds ratio. [a] Fully adjusted model controlled for sex, multiple birth, parental education, household income, parental depression and cognitive ability and parent-rated emotional symptoms at age 5. [b] OR_{XY} is the observed odds for the association between the exposure (X) and the outcome (Y).OR_{UX} is the odds between the exposure (X) and the unmeasured confounder (U) and OR_{UY} is the odds between the unmeasured confounder (U) and the outcome(Y). The maximum relative amount the unmeasured confounding could reduce an observed odds ratio is given by $B = OR_{UX}OR_{UY} / (OR_{UX}+OR_{UY}-1)$ and the minimum strength of OR_{UX} and OR_{UY} to have B explain away the observed odds ratio (OR_{XY}) is presented above. In order to determine the required minimum for both OR_{UX} and OR_{UY}, the two parameters are set equal to each other.
